# Editor’s Letter from Abroad

**Published:** 1873-09

**Authors:** 


HALL'S

### JOURNAL OF HEALTH.

Vol. XX.—SEPTEMBER, 1873.—No. IX.

### EDITOR'S LETTER FROM ABROAD.

At Sea, *June*, 1873.

When we came aboard the Cunarder *Java* the ladies were fanning themselves furiously, while the gentlemen were sweltering in perspiration. On the third day out the women were shivering in shawls, in the saloons, and the men, in their thickest overcoats, were affectionately hugging the smoke stack. Let this be a lesson to all sea-goers to take with them their mid-winter clothing, even in the hottest heats of summer. Ocean going steamers have first and second class passengers—the difference in fare being from twenty to fifty dollars, according to the locality of the berths. All eat at the same table, and at the same time. The outside berths are the best, being larger, better ventilated—having a long sofa to lie down upon without going into a berth ; but, as many are indisposed to eat much for a great part of the time, and for a great part of the way are in bed, the saving above named may be regarded as that much gained, and may be used to much greater advantage in seeing sights on land. Forty dollars, for example, will carry a man from London to Vienna, giving an opportunity of seeing a number of celebrated places: Havre, Paris, Cologne, Frankfort, Heidelberg, Dresden and others.

Among the two hundred passengers aboard there is but one celebrity. There are old men and young, women, children and girls. At first there seemed to be a disposition to hold aloof, each one from his neighbor, but in two or three days everybody seemed to be acquainted with every other body—and at table or on deck, everywhere, the greetings are those of old friends. Each day has its events, chief of which is, How many miles did we make the past day? meaning from noon of yesterday until noon of to-day. Thus far we have averaged over three hundred miles—that much nearer our journey's end. Sometimes, when the sea is smooth, the currents of the water in the ship's direction, and the wind favorable, a steamer will make nearly four hundred miles; and, taking the sailing distance between New York and Liverpool in round numbers—easily remembered at three thousand miles—a lessening of the distance of three or four hundred miles is a very comforting item.

The sight of another ship is of universal interest—always pleasurable; and then the questions, Where is she from? where is she going? what is the distance between us? with the feeling that her passengers are passing the same questions as to us.

A traveller finds himself constantly endeavoring to read the histories of his fellow travellers—in doing which we have no plain English letters and words; we have rather to deal with signs and symbols—as uncertain, sometimes, as the hieroglyphics of the times of the Pharaohs, when Rameses flourished and Job lived. Sometimes we read aright and sometimes wrong. Yet, from the dress, and manner, and bearing of a man, we soon learn where to place him. There is an easy distinction between the farmer, the parson, the lawyer and the country merchant—between a down east

Yankee and a man from the West. The first uttered word tells you whether your fellow passenger is from the north or from beyond Mason and Dixon's line, where the sugar cane grows, and where oranges are gathered, and cotton whitens the fields like a snow storm. There is one thing on board the *Java* which cannot fail to leave a pleasant impression in these degenerate days of Chicago divorces, and Indiana and Connecticut disgraces in the same line: it is the irresistible attention of gentlemen to their wives—their considerate care, the readiness with which they wait upon them—bringing them everything they ask for, and then, when they have made them as comfortable as possible, sitting by their side hour after hour, day and night, as if to be ready at a moment's notice to meet all their wishes. This has been observed between young and old, the plainly dressed and poor, as well as the merchant and the banker. These things speak well of the marriage relation among the great bulk of the people, and disprove effectually all the trash and trumpery of a noisy few, who seem to strive to make the world believe that there is not more than one "happy match" in a hundred married homes. There was one case of a gentleman of fifty and his sickly wife: he a handsome man, and she a poor, homely faced creature—both evidently persons of position at home—and yet the attentions of this man were quite as assiduous as those paid by a widower of forty-five to his queenly looking wife of twenty whom he has just married, this being their bridal tour. Akin to this is the beautiful fact that, of the hundreds lost in the steamer of the White Star line, there were no widows or widowers made; husbands and wives preferred to go down into a watery grave rather than abandon each other. We have now travelled many miles in the *Java* and have yet to hear the first loud or angry word, and not an oath has been uttered by passenger or sailor, no doubt owing to Captain Martin's influence—for, "like priest, like people," "like master, like crew;" and where the deportment of the commander is quiet, composed, self-respecting, its spreads its restraining influence over all parts of the ship.

It was beautiful, on Sunday morning, to be a spectator at the religious services. Nearly every passenger was in the saloon, and, punctually at the appointed hour, the sailors who could be spared filed in, in their best clothing, and reverently took their places with bowed heads. The services of the Episcopal Church were performed by Captain Martin in a most appropriate manner; every syllable and every word—not excepting the proper names—all were as accurately and as distinctly pronounced as if the reader had been a graduate of Yale or Harvard, and with an appropriateness of manner and tone which made the beautiful service both impressive and useful—all preparing us for a subsequent remark of our captain in a casual conversation: "The American celebrates the Fourth of July and the Briton the birthday of her Gracious Majesty the Queen, and it is well that the triumphs of both nations should be perpetuated in our remembrances; but the triumphs of Christianity merit a greater, and a truer, and a more glorious recognition." After a pleasant and smooth passage we came in sight of Queenstown, the seaport of Cork, a dozen miles further up the River Lee, by rail or boat. Generally steamers from America agree to land passengers at Queenstown, but they are allowed to carry them on to Liverpool, eighteen hours further on, if the weather is not favorable. Nor do the ships "land" at Queenstown at all. Small tug boats meet them ten miles out at sea, which take off the mails and passengers while the steamer goes on her way. This ten miles of "tugging" is anything but agreeable, even in the best of weather; but at night, or in a rain, or high sea, it is the next thing to crossing between England and France. Yet many prefer to land at Queenstown as it shortens the passage a day or two, while Liverpool is reached in eight hours by rail. It does not require a steamer two days to run from Queenstown to Liverpool; the vessel may arrive at the latter place in eighteen hours, but then it may be at low tide, and there must be a waiting of twelve hours longer. But if persons wish to travel through Ireland, to see the Groves of Blarney and the Lakes of Killarney, passing on to Dublin, Belfast and the Giant's Causeway, it is a saving of time and money to land at Queenstown, where Wolfe was buried, who made himself immortal, and Sir John More, too, from having written a piece of inferior poetry about a man who lost a battle.

